# Recent Development in University Student Learning Research in Blended Course Designs: Combining Theory-Driven and Data-Driven Approaches

**DOI:** 10.3389/fpsyg.2022.905592

**Published:** 2022-05-17

**Authors:** Feifei Han

**Affiliations:** ^1^Department of Pedagogy and Psychology, Faculty of Education, The University of Hradec Králové, Hradec Králové, Czechia; ^2^Griffith Institute for Educational Research, Griffith University, Brisbane, QLD, Australia

**Keywords:** university student learning research, blended course designs, theory-driven approaches, data-driven approaches, a combined approach

## University Student Learning in Blended Course Designs

Advances in Internet and computer-based technologies have increased the growth of alternative learning spaces, creating entirely new ways of conceptualizing and assessing the learning experience of students (Wong, 2019). Research has repeatedly found positive impacts of various forms of technology-enhanced learning, such as learning embedded in social networking sites, web-conferencing, webinars, e-portfolio, digital games, mobile apps, and virtual and second worlds (Jarvoll, 2018; Winkelmann et al., 2020). The higher education sector has also shown a rapid development in the use of computer and web-based technologies, which has affected experiences of learning of a significant proportion of university students worldwide. This change has resulted in the widespread use of diverse online spaces developed for learning in the form of e-learning courses and/or a combination of face-to-face and online delivery systems, known as blended course designs (Shin et al., 2018). In a synergy of research foci and research methods of university student learning in blended contexts, Bliuc et al. (2007) defines blended course designs as “systematic combination of co-present (face-to-face) interactions and technologically-mediated interactions between students, teachers and learning resources” (p. 234).

More recently, the coronavirus pandemic (COVID-19) emergency has required higher education learning and teaching around the world to rapidly respond, in particular, redeploying even more learning and teaching activities to virtual learning spaces to promote physical distancing. As a result, more and more face-to-face courses have been delivered as blended courses (Mali and Lim, 2021). Under such circumstances, it becomes vital importance to understand student learning experience in blended course designs, its relation to various forms of learning outcomes, and the key factors which may impact such experience. However, there are many challenges to study the student experience in blended course designs as it is a complex phenomenon (Ma and Lee, 2021). Compared with learning in a single mode (i.e., fully face-to-face courses or fully online courses), in blended course designs, students are increasingly involved in decision-making in the learning process, such as with whom to work in a classroom tutorial and with whom to discuss in online forum, how many hours they learn online, whether to study in a physical library or log onto an online database, whether to collaborate in a face-to-face laboratory or whether to collaborate in virtual reality. These decisions require students to move back and forth between face-to-face and online contexts, and across physical and virtual learning environments (Han and Ellis, 2020a). As a result, the complexity of student experience in blended learning involves an interplay of a wide range of factors, which include students' cognition (e.g., conceptions, approaches, and perceptions in learning) (Trigwell and Prosser, 2020); their choices of social interactions in learning (e.g., with whom to collaborate and the mode of the collaborations) (Hadwin et al., 2018); and the material elements across both physical and virtual learning spaces in face-to-face and online learning components (e.g., students' choices of learning spaces and their interactions with online learning activities) (Laurillard, 2013). As such, evaluation of university student learning experience in blended course designs requires research methods that move beyond approaches that do not routinely investigate the combined contributions of learners and the material things involved in learning to achieve academic performance, (Wu et al., 2010; López-Pérez et al., 2011).

## Theory-Driven Approaches

Traditionally, research into student learning experience and academic performance in higher education has largely adopted theory-driven approaches, which test hypotheses derived from theories in educational psychology, learning sciences, and pedagogy and curriculum research (Trigwell and Prosser, 2020). Based on the accumulated empirical evidence which may support or refute the hypotheses, theories and models of learning have been constantly refined, modified, and updated. The theoretical frameworks departing from theory-driven perspectives predominantly assess various aspects in student learning experience using self-reported instruments and measurements, such as focus group, semi-structured interviews, and Likert-scale questionnaires (Han et al., 2022).

In a number of state-of-the-art articles, for instance, the instruments in the major frameworks on the student learning research in higher education have been reviewed and summarized, including Student Approaches to Learning Research, Self-regulated Learning Research, Information Processing Research, and Student Engagement Research (Lonka et al., 2004; Vermunt and Donche, 2017; Zusho, 2017). Uniformly, the primary means to collect data in these frameworks are self-reported questionnaires, such as Study Process Questionnaire (SPQ; Biggs et al., 2001), the Revised Approaches to Studying Inventory (RASI; Entwistle and McCune, 2004), and the Inventory Learning to Teach Process (ILTP; Endedijk et al., 2016) and Motivated Strategies for Learning Questionnaire (MSLQ; Pintrich, 2004); Inventory of Learning Patterns (ILP; Donche and Van Petegem, 2008), Course Experience Questionnaire (CEQ; Ramsden, 1991), and the Inventory of Perceived Learning Environments Extended (IPSEE; Könings et al., 2012).

However, the self-reported measures have been criticized for being subjective and have been questioned about their accuracy in describing students' use of learning approaches and strategies in real learning contexts (Zhou and Winne, 2012). In addition, the self-reported measures and data also suffers from their limited capacities to represent the complex (e.g., using multiple indicators) and dynamic (e.g., changes over time) nature of student learning behaviors. To improve the insights of contemporary university student experiences of learning, suggestions have been put forward to expand the current self-reporting methods by including other types of measurements to study student learning (Vermunt and Donche, 2017). In this regard, learning analytics research is a promising avenue. For instance, Richardson (2017) suggested “The rapidly expanding field of learning analytics provides both researchers and practitioners with the opportunity to monitor students' strategic decisions in online environments in minute detail and in real time (p. 359).”

## Data-Driven Approaches

The recent development of educational technology has produced prolific studies using learning analytics, which enables a capacity to collect rich and detailed digital traces of students' interactions with a variety of online learning resources and activities. The type of digital trace/log data, also known as the observational data, have the advantage of offering descriptions of student learning behaviors and strategies relatively more objectively and in a more granular details than using self-reported methods (Siemens, 2013; Baker and Siemens, 2014). Departing from data-driven approaches, learning analytics research has emerged as a growing area and has gradually gained popularity in student learning in higher education (Sclater et al., 2016). It employs advanced data mining techniques and algorithms to process the observational analytic data in relation to students' demographic information, which has been increasingly applied in various domains in higher education sector, such as advising students' career choice (Bettinger and Baker, 2013); detecting at risk students to improve retention (Krumm et al., 2014); providing personalized feedback (Gibson et al., 2017); identifying patterns of learning tactics and strategies (Chen et al., 2017); facilitating collaborative learning (Kaendler et al., 2015); monitoring students' affect in learning (Ocumpaugh et al., 2014); and predicting their academic learning outcomes (Romero et al., 2013). However, the data-driven approaches are often fragmented from educational theories and rely purely on empiricism, which limit the insights they can offer for directing pedagogical innovations and reforms, supporting learning design, fostering quality learning experience, and improving academic performance (Buckingham Shum and Crick, 2012).

Despite its wide applications, learning analytics research have received criticism that the data-driven approaches it relies on is fragmented from educational theory but overly focuses on quantitative number (Rodríguez-Triana et al., 2015). The patterns and models of student learning derived from such data-centric perspectives without proper guidance from educational theories are often result in erroneous interpretation, which have limited insights for generating actionable knowledge in order to locate learning barriers and to offer ideas for teaching practice and curriculum design (Wong and Li, 2020; Han and Ellis, 2021).

## A Combined Approach

Recognizing the limitations of an overly data-centric approach used in learning analytics research, researchers have proposed to adopt a more holistic approach when designing research so that student learning behaviors and patterns can be captured in a comprehensive manner, and big data modeling and interpretation can be guided via sound theories (Lockyer et al., 2013; Rienties and Toetenel, 2016). This has resulted in an increasing amount of research using a combined approach, which employs both self-reported and observational instruments to measure student learning experience in a complementary manner (Gašević et al., 2015).

Reviewing the existing research using a combined approach suggests that these studies have two different aims. One aim is to examine how a combined approach may improve the explanatory power of predicting student learning outcomes by including both self-reported and observational measures of aspects in the processes of student learning (Reimann et al., 2014). Despite basing on different learning theories, the majority of studies in this line of inquiry have reported that combing the self-reported and observed measures of student learning have significantly improved the variance explained in the prediction of student academic achievement than using either self-reporting or observational method alone (Tempelaar et al., 2015; Han and Ellis, 2020b). For example, using multiple regression analyses, Pardo et al. (2017) found that students' reported anxiety in learning and use of self-regulated learning strategies could only explain 7% of variance in students' academic performance; whereas adding the observed frequency of students' online interactions into the regression model could explained 32% of variance in students' academic performance, significantly increasing the total variance explained by 25%. In another study which adopted Student Approaches to Learning research framework, Ellis et al. (2017) reported similar findings: while students' self-reported use of approaches to learning only predicted 9% of the variance in their academic learning outcomes, including students' observed online learning events in the multiple regression equation explained an extra of 25% in the students' learning outcomes.

Another aim of the studies which combine the self-reported and observational measures is to investigate the consistency between the two methods in terms of describing student experiences of learning (Reimann et al., 2014). Similarly, research with this aim has also departed from different learning perspectives and has diverse foci, such as using self-reported questionnaires to assess students' intrinsic motivation, test anxiety, self-efficacy, engagement, effort expenditure, achievement goal, learning orientations and motives on the one hand. On the other hand, a diversity of observed indicators of students' online learning, including frequency of clicks, completion of online learning tasks, duration of online learning events, as well as time-stamped sequences of online learning behaviors have been collected through digital traces to derive students' online learning tactics, strategies, and approaches (Gašević et al., 2017; Pardo et al., 2017; Han et al., 2020; Sun and Xie, 2020; Ober et al., 2021). However, inconclusive results have been reported among studies with this aim. The non-alignment between the descriptions and categorisations of student learning experience by self-reports and observations have been found in a number of studies.

For instance, Gašević et al. (2017) found that there was no significant difference of self-reported using surface learning strategies between students who were categorized as deep and surface learners according to their observed online learning strategies. In contrast, consistency between students' self-reported learning orientations (as measured by their approaches to learning and perceptions of learning) and their online participation was observed in Han et al. (2020). Students who were categorized as having an “understanding” learning orientation (i.e., the learning was oriented toward gaining an in-depth understanding of the subject matter) were also observed to participate more online learning than those who were categorized as having a “reproducing” learning orientation (i.e., the learning was oriented toward reproducing facts and satisfying course requirements).

These inconsistent results are possibly caused by (1) different learning theories have been adopted by these studies (e.g., self-regulated learning, Student Approaches to Learning, student engagement); and (2) the diverse means of how students' online learning is measured (e.g., frequency, duration, temporal sequence, or a combination of multiple indicators). Until a firmer conclusion can be made, many more studies are required to examine the extent to which the self-reported and observational measures of student learning experience align with each other.

The strengths of a combined approach lie in multiple ways. First, it has an advantage of offering richer information in the way of predicting student learning outcomes over using a single approach, with each approach supplementing the other. While the observational measures are able to provide objective evidence as to what students actually do in their learning (Fincham et al., 2018), they do not, however, have capacity to reflect students' intents and motives behind the ways they learn as in the self-reported studies (Asikainen and Gijbels, 2017; Gerritsen-van Leeuwenkamp et al., 2019). Second, a combined approach can serve as a triangulation to check the validity of the results derived from either theory-driven or data-driven approaches. Third, the multiple data collection and analysis methods used in the combined approach also strengthen the analytical power of the analyses. All the merits of combining theory-driven and data-driven approaches point out its future applications to advance university student learning research and its potential to tackle the other complex issues of contemporary student experiences of learning.

## Author Contributions

FH contributed to the conception of the work, drafted the work and revised it critically for important intellectual content, approved the final version of the paper to be published, and agreed to be accountable for all aspects of the work in ensuring that questions related to the accuracy or integrity of any part of the work are appropriately investigated and resolved.

## Funding

This study was supported by the International mobilities for research activities of the University of Hradec Králové II, CZ.02.2.69/0.0/0.0/18_053/0017841.

## Conflict of Interest

The author declares that the research was conducted in the absence of any commercial or financial relationships that could be construed as a potential conflict of interest.

## Publisher's Note

All claims expressed in this article are solely those of the authors and do not necessarily represent those of their affiliated organizations, or those of the publisher, the editors and the reviewers. Any product that may be evaluated in this article, or claim that may be made by its manufacturer, is not guaranteed or endorsed by the publisher.
